# Malignancy rates for Bethesda III and IV thyroid nodules: a retrospective study of the correlation between fine-needle aspiration cytology and histopathology

**DOI:** 10.1186/s12902-020-0530-9

**Published:** 2020-04-15

**Authors:** Busra Yaprak Bayrak, Ahmet Tugrul Eruyar

**Affiliations:** Department of Pathology, Faculty of Medicine, Kocaeli University, 41380 Kocaeli, Turkey

**Keywords:** Fine-needle aspiration cytology, Thyroid nodule, Thyroidectomy, Malignancy rate

## Abstract

**Background:**

Fine-needle aspiration cytology (FNAC) has become a well-established modality in the diagnosis, staging and follow-up of thyroid nodules. FNAC outcomes are routinely classified using the Bethesda System for Reporting Thyroid Cytopathology (BSRTC), facilitating appropriate clinical management. Bethesda categories III and IV encompass varying risks of malignancy. This retrospective study established a possible association between these cytological categories and malignancy rates in patients treated at a single institution.

**Methods:**

Over a 6-year period, 11,627 FNAC procedures were performed on thyroid nodules. Of these, 814 (59.63%) patients were submitted to thyroidectomy. The nodules of 108 patients were classified as Bethesda category III and 47 patients as Bethesda category IV. Patient data were reviewed to establish a correlation between the FNAC results and the final histopathological analyses.

**Results:**

The rates of malignancy among patients who underwent surgery were 25% for category III and 27.6% for category IV, with no significant differences between categories (*p* = 0.67). The pathological parameters of malignant nodules, namely tumour type, size, encapsulation, invasion into the thyroid capsule, extrathyroidal extension and lymphovascular invasion did not significantly differ between the groups (*p* > 0.05).

**Conclusions:**

This paper provides a more precise correlation of malignancy rates with thyroid nodules classified as Bethesda categories III and IV, as our findings are comparable to the literature, giving malignancy rates ranging from 10 to 30% for category III and 25–40% for category IV. Use of the BSRTC is heterogeneous across institutions, and there is some degree of subjectivity in the distinction between categories III and IV; therefore, it is crucial to estimate the rates of malignancy at each institution. Molecular assays are of increasing importance in determining the need for surgical intervention for thyroid lesions. Gene expression assays using FNAC material may demonstrate a high predictive value for cytologically indeterminate thyroid nodules diagnosed as Bethesda classes III and IV.

## Background

Fine-needle aspiration cytology (FNAC) has become a well-established diagnostic technique. It accelerates the assessment of cellular morphologic features of thyroid nodules from which the malignant risk can be determined. This information is important when planning the therapeutic management of nodules, deciding in follow-up of the nodule size, repeating the biopsy or performing a total or partial thyroidectomy [1, 2]. Although FNAC is widely used in clinical diagnosis, cytologically indeterminate thyroid nodules continue to present a diagnostic challenge for pathologists. This makes reaching a definitive histologic diagnosis difficult in a large number (10–30%) of patients undergoing thyroidectomy [3].

Since 2009, The Bethesda System for Reporting Thyroid Cytopathology has been used to classify FNAC findings based on the risk of malignancy [4, 5]. There are six cytological diagnostic categories, each with different suggested treatment approaches. Bethesda categories II, V and VI are well established, and therefore not subject to any disagreement in terms of their malignancy rates [6]. However, there are controversial data about the risk of malignancies, recurrence and clinical management of nodules in Bethesda categories III and IV, as the reported risks of malignancy vary significantly, from 10 to 30% to 25–40% (including noninvasive follicular thyroid neoplasm with papillary-like nuclear features [NIFTP]), respectively [4]. This also leads to different approaches to choosing the best therapies. Bethesda category III describes the cytological findings as “atypia of undetermined significance” (AUS) and “follicular lesion of undetermined significance” (FLUS), while Bethesda category IV represents “follicular neoplasm/suspicious for follicular neoplasm” (FN/SFN) [1, 4–6]. The FN/SFN category presents the greatest uncertainty, as follicular carcinomas resemble benign follicular neoplasms at the individual cellular level, hence limiting the ability of pathologist to accurately diagnose these nodules unless the tissue demonstrates any vascular or capsular invasion [7]. The other known cytological category of AUS/FLUS covers a subset of lesions that are not easily classified as benign, suspicious or malignant [4]. A crucial advantage of the Bethesda III category is that FNAC specimens may need to be reevaluated, and in the case of a suspected follicular carcinoma, rebiopsy and operative intervention should be considered [4].

The difficulty in defining the exact diagnosis of thyroid nodules is underlined by the fact that the probability of malignancy in AUS/FLUS or FNAC specimens remains unclear [4, 8, 9]. Some malignancy criteria such as thyroidal or tumoral capsular and/or lymphovascular invasion are determinative when establishing a cancer diagnosis, which represents a significant limitation of the FNAC method. Considering these limitations and debates on the management of Bethesda III and IV thyroid nodules, together with the diverse malignancy rates reported in the literature, the present retrospective study aimed to attribute an accurate malignancy rate for patients with nodules classified as Bethesda III or IV. We also aimed to establish whether there is an association between these cytological categories and malignancy rates in patients, based on data collected over 6 years at a single institution.

## Methods

### Patients

From January 2012 to July 2017, 11,627 FNAC procedures were performed for thyroid nodules. A total of 814 (59.63%) of these patients underwent thyroidectomy. The age of patients at the time of operation ranged from 18 to 86 years. The gender distribution showed a female preponderance, with 664 females and 150 males. The selection criteria for the study were patients with thyroid nodules who underwent FNAC as the primary diagnostic modality followed by total or partial thyroidectomy. After clinical and radiological diagnosis, the FNA procedure was performed under ultrasound guidance. Patients presenting thyroid nodules with a cytological analysis suggestive of Bethesda classes I, II, V and VI were excluded from the evaluation, along with those diagnosed with Bethesda III and IV with no follow-up data.

### Cytology

The cytopathological reports were issued by a pathologist, following the Bethesda classification according to the literature [1, 4]. In our thyroid FNAC practice, the Bethesda III category was divided into AUS and FLUS. AUS was defined as cases with follicular cells that were mostly benign in appearance with rare nuclear atypia, while FLUS was defined as cases with extensive Hurthle cells with moderate cellularity, scant colloid with no apparent increase in lymphoid cells, and follicular epithelial cell clusters showing a microfollicular pattern in the focal area. Aspirations were performed according to the literature [8]. Smears were either air-dried and stained with May-Grünwald-Giemsa stain without fixation, or fixed with alcohol then stained with Papanicolaou stain.

The medical records of each patient were reviewed to establish an association between the FNAC results and the final histopathological diagnosis. The exact position of the nodule in the gland, the final histopathological analysis of the target nodule and other pathologic findings were considered to confirm that the cytology and histopathology results were for the same nodule.

### Histology

All thyroid tissues were fixed in 10% neutralised formaldehyde. Nodules suspected for malignity were totally embedded in paraffin, and stained with haematoxylin and eosin (H&E).

Histological analysis was performed on all surgically excised lesions that were the target of cytological evaluation. The FNAC results were compared with histopathology as the gold standard method.

### Statistical analysis

GraphPad version 3.062003 software was used for statistical analyses. The nonparametric Mann-Whitney test was used to compare quantitative variables, while the chi-square test or chi-square test for independence were used to compare dependent or independent qualitative data. A *P*-value less than 0.05 was considered significant.

## Results

**Fig. 1:**
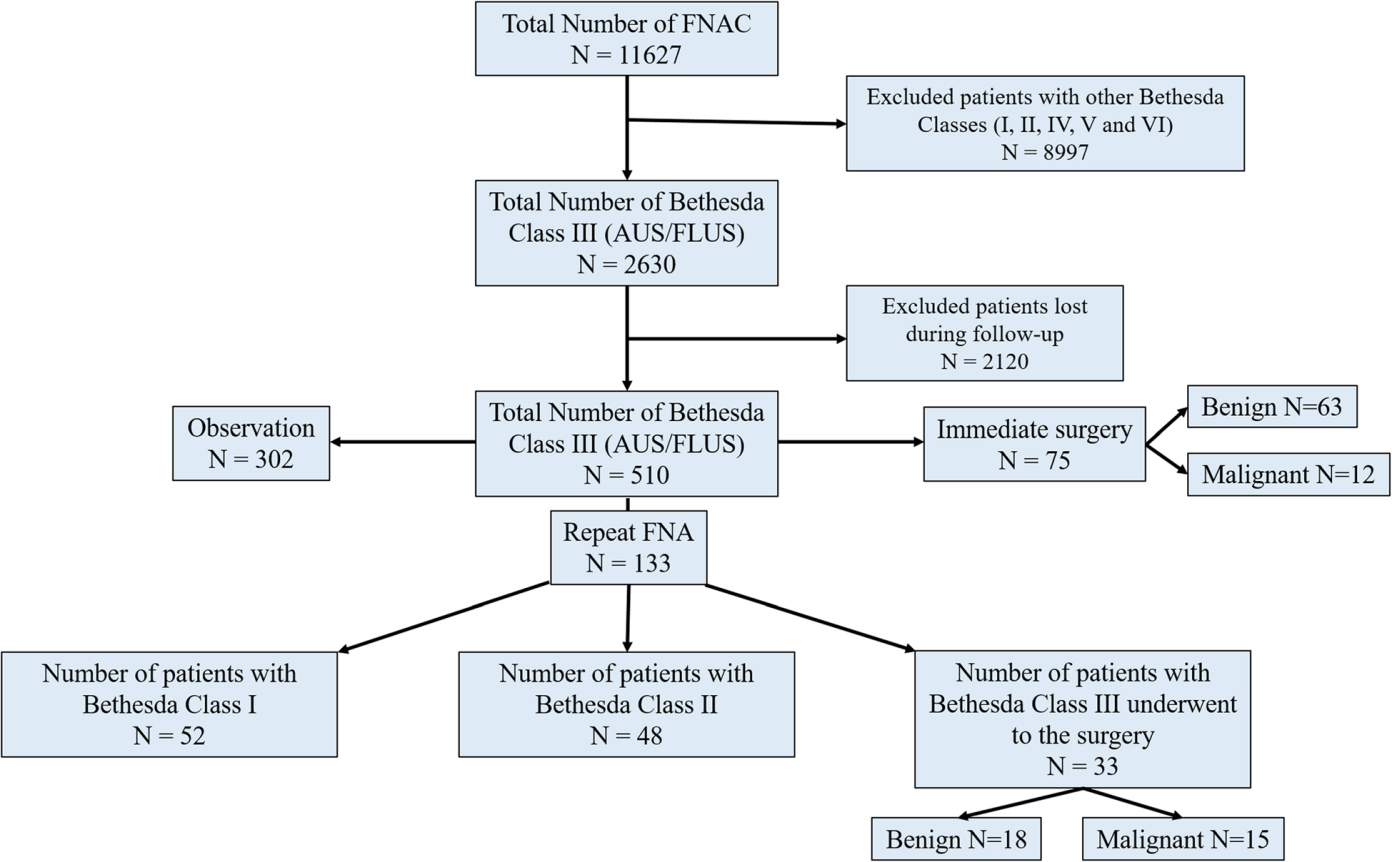
Flow chart of the number of fine-needle aspiration cytology (FNAC) procedures on thyroid nodules leading to a diagnosis of atypia of undetermined significance/follicular lesion of undetermined significance (AUS/FLUS)

**Fig. 2:**
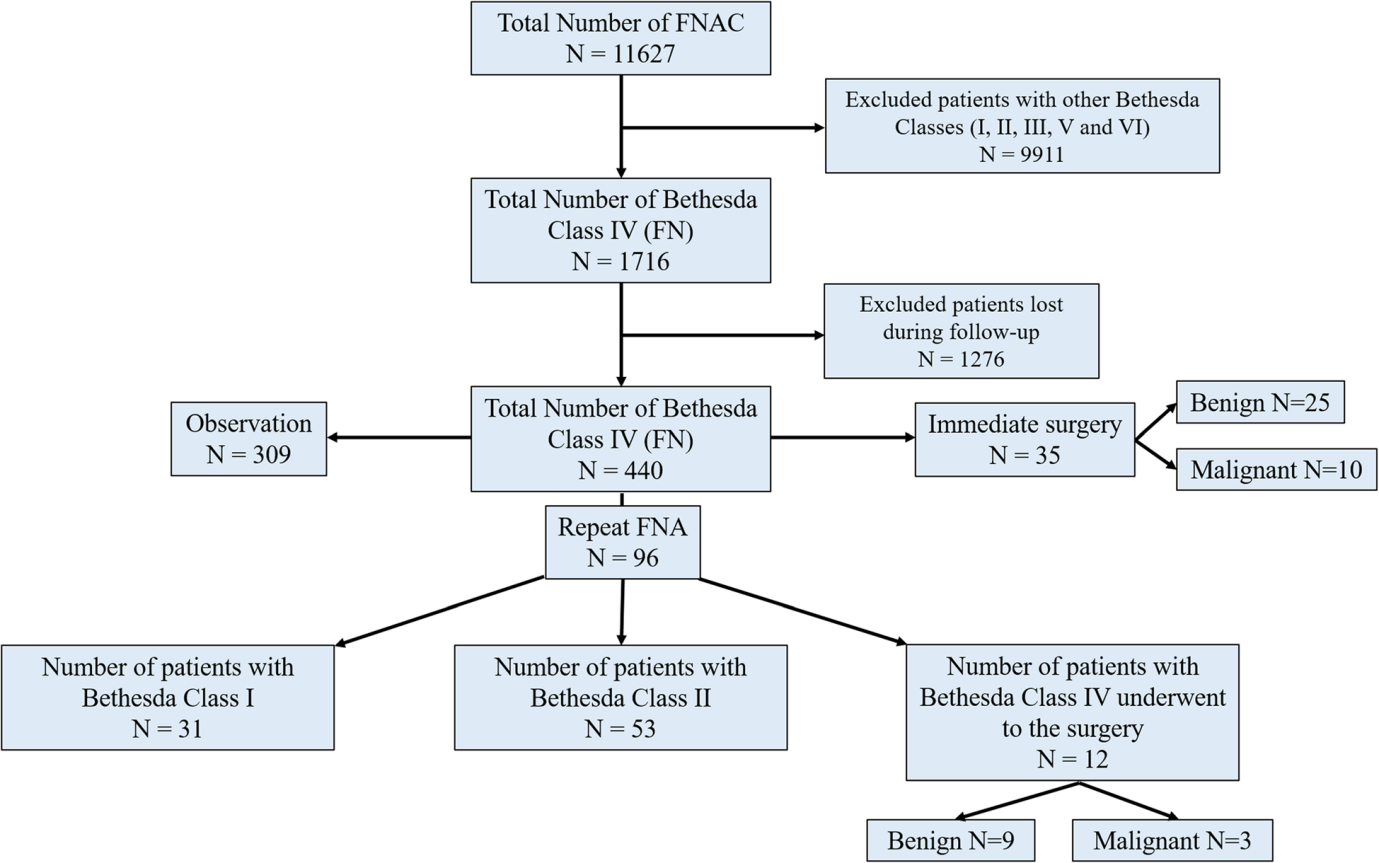
Flow chart of the number of fine-needle aspiration cytology (FNAC) procedures on thyroid nodules leading to a diagnosis of follicular neoplasm/suspicious for follicular neoplasm (FN/SFN)

Overall, 4.2% (2630/11627) of all thyroid FNAs performed during the study period were classified as AUS/FLUS (Fig. 1) and 6.8% (1716/11627) were classified as FN/SFN (Fig. 2), in accordance with the Bethesda System for Reporting Thyroid Cytopathology guidelines. Of the 2630 patients diagnosed with AUS/FLUS on initial FNAC, 510 (19.4%) were documented during follow-up. Of 1716 patients with FN/SFN on initial FNA, 440 (2.6%) were documented during follow-up.

Among the cases classified as Bethesda category III (*n* = 510), 75 (14.7%) underwent immediate surgery, 133 (26.1%) underwent repeat FNAC in 1–3 months, and 302 (59.2%) underwent ultrasonography monitoring at 3-month intervals to measure the size and content of the nodule. Of the 133 nodules that required repeated FNAC, 52 (39.1%) were identified as Bethesda class I, 48 (36.1%) as Bethesda class II and 33 (24.8%) as class III. All patients with nodules with two consecutive AUS/FLUS diagnoses (*n* = 33) underwent surgery, of which 45.5% (15/33) were found to be malignant while 54.5% (18/33) were benign (Fig. 1).

Among the cases in Bethesda category IV (*n* = 440), 35 (8.0%) underwent immediate surgery, 96 (21.8%) underwent repeat FNAC in 1–3 months, and 309 (70.2%) were observed at 3-month intervals via ultrasonography to measure the size and content of the nodule. Of the 96 nodules that required repeat FNAC, 31 (32.3%) were identified as Bethesda class I, 53 (55.2%) as Bethesda class II and 12 (12.5%) as class IV. All patients with nodules with two consecutive FN/SFN diagnoses (*n* = 12) underwent surgery, of which 75% (9/12) were found to be malignant while 25% (3/12) were benign (Fig. 2).

**Fig. 3:**
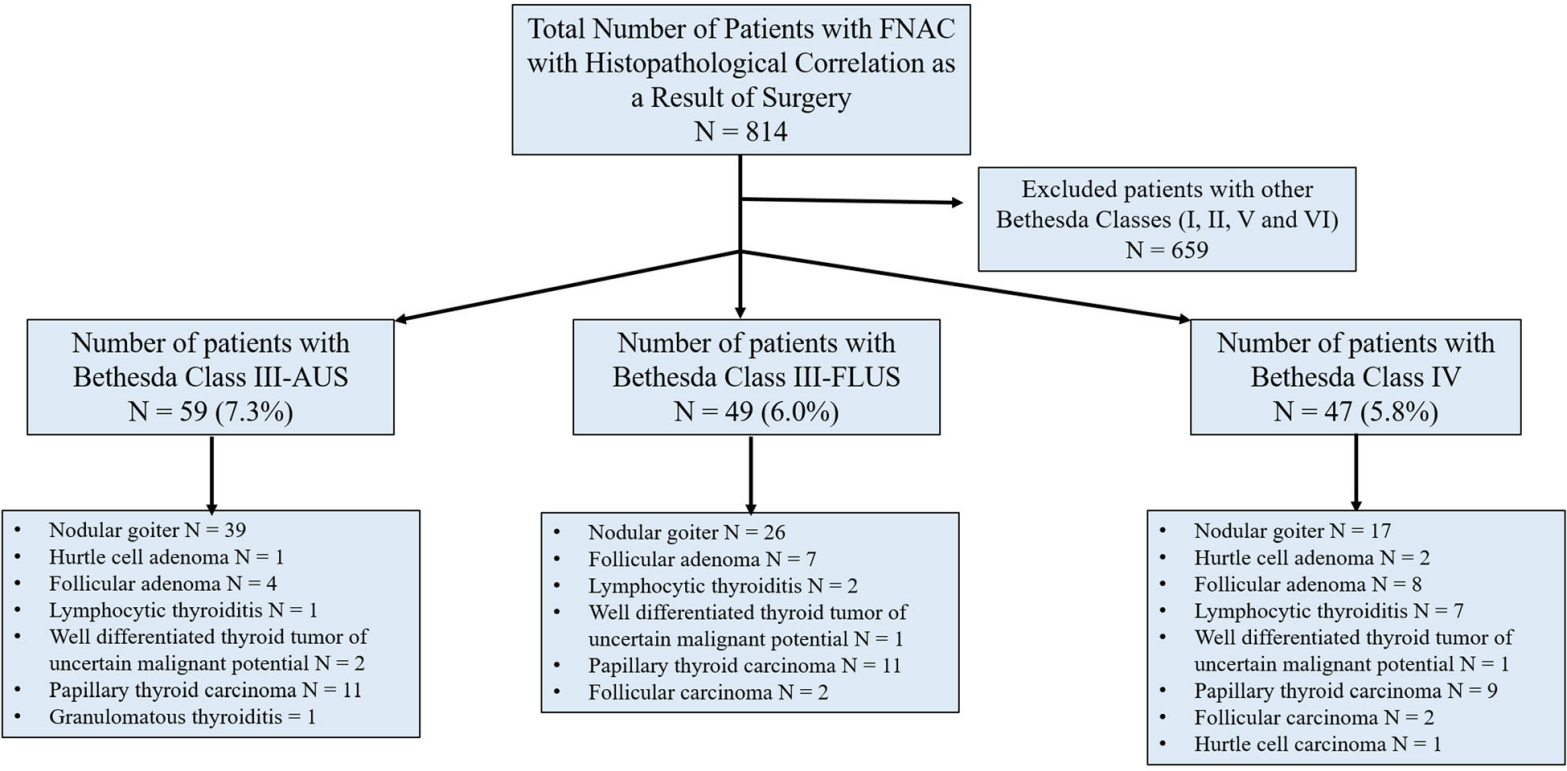
Flow chart of the number of fine-needle aspiration cytology (FNAC) procedures on thyroid nodules leading to a diagnosis of Bethesda class III (atypia of undetermined significance [AUS] or follicular lesion of undetermined significance [FLUS]) or class IV (follicular neoplasm/suspicious for follicular neoplasm [FN/SFN])

The 155 patients with nodules diagnosed by FNAC followed by resection presented with Bethesda category III or IV. Among them, 108 were diagnosed with AUS/FLUS (59 patients were AUS and 49 were FLUS) and 47 were diagnosed with FN/SFN (Fig. 3). The majority of patients were female (85.2%) and 13.8% were male. The mean age of patients was 52.5 ± 1.0 years (Table 1). When comparing the localisation of nodules in the AUS/FLUS and FN/SFN groups, nodules in both groups were more frequently located in the right lobe of the thyroid (60.2 and 61.7%, respectively). The least frequent location of nodules was the isthmus (2.8% in the AUS/FLUS group and 8.5% in the FN/SFN group; Table 1).

**Table 1:** Demographic features of AUS/FLUS and FN cases diagnosed by FNAC following the resection

Characteristics	AUS/FLUS	FN	Total
Number (N)	108	47	155
Age (Mean ± SEM)	51.4 ± 1.1	54.5 ± 2.1	52.5 ± 1.0
Gender (N)			
Male (%)	15 (13.8)	8 (17.0)	23 (13.8)
Female (%)	93 (86.2)	39 (83.0)	132 (85.2)
Localization (N)			
Right (%)	65 (60.2)	29 (61.7)	94 (60.7)
Left (%)	40 (37.0)	14 (29.8)	54 (34.8)
Isthmus (%)	3 (2.8)	4 (8.5)	7 (4.5)

*AUS* Atypia of undetermined significance

*FLUS* Follicular lesion of undetermined significance

*FN* Follicular neoplasia

### Risk of malignancy among patients with AUS/FLUS nodules triaged to surgery

For the 75 (14.7%) patients with nodules classified as AUS/FLUS who underwent immediate surgery, the rate of malignancy was 16% (12/75). Patients with two successive FNAC tests showing AUS/FLUS had a malignancy rate of 45.5% (15/33), with benign nodules representing 54.5% (18/33; Fig. 1). Including the 33 nodules that were resected (after repeat FNAC), the rate of malignancy for all patients triaged to surgery was 25% (27/108; Table 2).

**Table 2:** Malignancy ratios of thyroid nodules diagnosed in Bethesda III and IV classes following the resection.

	Benign N (%)	Malignant N (%)	Total	*P* value
Bethesda III	81 (75.0)	27 (25.0)	108	0.67^a^
AUS	46 (78.0)	13 (22.0)	59	
FLUS	35 (71.4)	14 (28.6)	49	
Bethesda IV	34 (72.3)	13 (27.6)	47	

*AUS* Atypia of undetermined significance

*FLUS* Follicular lesion of undetermined significance

^a^Chi-square Test compared Bethesda III and IV groups

### Risk of malignancy among patients with FN/SFN nodules triaged to surgery

For the 35 (8.0%) patients with nodules classified as FN/SFN who underwent immediate surgery, the rate of malignancy was 28.6% (10/35). Patients with two successive FNAC tests showing FN/SFN had a malignancy rate of 25% (3/12) and benign rate of 75% (9/12; Fig. 2). Including the 12 nodules that were resected (after repeat FNAC), the rate of malignancy for all patients triaged to surgery was 27.6% (13/47; Table 2).

### Comparison of malignancy rates between Bethesda classes

Malignancy was diagnosed in 25% of 108 patients in Bethesda group III and 27.6% of 47 patients in Bethesda group IV (Table 2). There was no statistical difference between AUS, FLUS and FN/SFN groups in terms of malignancy rates (*P* = 0.67). Regarding histopathological findings, benign lesions included nodular goitre, Hurtle cell adenoma, follicular adenoma, granulomatous thyroiditis and lymphocytic thyroiditis. In comparison, histopathologically malignant lesions included well-differentiated thyroid tumours of uncertain malignant potential, papillary thyroid carcinoma, follicular carcinoma and Hurtle cell carcinoma (Fig. 3). Among the malignant lesions, the most frequently diagnosed entity was papillary thyroid carcinoma, diagnosed in 81.5% of AUS/FLUS and 69.2% of FN/SFN patients (Table 3). There was no significant difference between groups in terms of tumour type (*P* = 0.65). The average size of malignant tumours was 1.91 ± 0.15 cm, with no difference between groups (*P* = 0.78). Three patients in the AUS/FLUS group had encapsulated tumours, while none of the FN/SFN patients had encapsulation. The rate of invasion into the thyroid capsule was higher in the FN/SFN group (46.2%) compared to the AUS/FLUS group (22.2%), although there was no significant difference between groups (*P* = 0.24). Also, the parameters of extrathyroidal extension (defined as extension of the primary tumour outside the capsule and invasion into the surrounding tissue) and lymphovascular invasion did not differ significantly between the groups (*P* = 0.97 for both parameters).

**Table 3:** Pathological parameters of malign thyroid nodules diagnosed in Bethesda III and IV classes following the resection.

	AUS/FLUS (*N*=27)	FN (*N*=13)	Total (*N*=40)	*P* value
Tumor type				
Follicular carcinoma	2 (7.4%)	2 (15.4%)	4 (10%)	0.65^a^
Papillary thyroid carcinoma	22 (81.5%)	9 (69.2%)	31 (77.5%)	
Others	3 (11.1%)	2 (15.4%)	5 (12.5%)	
Tumor size (cm of the largest nodule)	1.97 ± 0.19	1.77 ± 0.20	1.91 ± 0.15	0.78^b^
Encapsulation	3 (11.1%)	0	3 (75%)	0.54^c^
Tumor invasion into thyroid capsule	6 (22.2%)	6 (46.2%)	12 (30%)	0.24^c^
Extrathyroidal extension	2 (7.4%)	1 (7.7%)	3 (75%)	0.97^c^
Lymphovascular invasion	2 (7.4%)	1 (7.7%)	3 (75%)	0.97^c^

*AUS* Atypia of undetermined significance

*FLUS* Follicular lesion of undetermined significance

*FN* Follicular neoplasia

^a^Chi-squared Test for Independence

^b^Nonparametric Mann-Whitney Test

^c^Chi-square Test

## Discussion

Reporting of FNAC results has been successfully standardised by the Bethesda System for Reporting Thyroid Cytopathology, which also facilitates more accurate diagnostic decisions in clinical management. The aim of this categorisation system was to achieve a multidisciplinary consensus and to clarify the malignancy rates of lesions in different classes. However, the absolute level of risk and malignancy is still unclear for thyroid nodules assigned to Bethesda categories III and IV [10, 11]. The 4th edition of the WHO Classification of Tumors of Endocrine Organs, published in 2017, introduced borderline tumours (uncertain malignant potential [UMP] and NIFTP) into thyroid tumour classification [12]. However, these tumours, which are characterised as invasive (papillary thyroid carcinoma [PTC]), incomplete invasive (well-differentiated thyroid tumour [WDT-UMP]) and noninvasive (NIFTP), were still classified as malignant tumours of the intrathyroidal encapsulated follicular variant (EFV) PTC in the 2015 American Thyroid Association (ATA) guidelines. Our laboratory was following the ATA principles during the period of data collection for this study (2012–2017); therefore, among the malignant cases, three patients with WDT-UMP (11.1%) in Bethesda group III and one case (7.7%) in Bethesda group IV were considered at risk of malignancy [13, 14]. There were no cases of NIFTP among our thyroidectomy patients.

Although some researchers argue that it would be useful to eliminate or reduce the categories for diagnostic cytopathology, Shi et al. found that eliminating AUS/FLUS significantly decreased the sensitivity of FNAC and increased the rates of false positive and false negative results [11]. The present study analysed the cytopathological findings of thyroid nodules of 950 patients at a single institution, classified into two categories: AUS/FLUS or FN/SFN. Based on histology, 510 of the FNAC specimens were classified into the AUS/FLUS category while 440 were in the FN/SFN category. Some series report an AUS/FLUS diagnosis rate of 18% among cytopathological specimens [15]; however, Ho et al. reported an incidence of AUS/FLUS diagnoses of 8% [8]. Compared to these previous findings, we report a higher rate of AUS/FLUS cases (22.6%) while the rate of FN/SFN cases was 14.8%. It should be mentioned that the number of patients diagnosed with AUS/FLUS and FN/SFN in the current study was limited. Furthermore, predicting the exact risk of malignancy in undetermined thyroid nodules is limited in that not all resected nodules undergo histopathologic analysis. Another limitation of this study was the loss of patients to follow-up over the 6-year period, as many patients were transferred to another university hospital or another surgeon [16].

The rates of malignancy for Bethesda III and IV nodules may vary among institutions, and they are likely to be higher in multicentre studies. In the literature, the malignancy rates for tumours in Bethesda categories are approximated as 10–30% for AUS/FLUS and 25–40% for FN/SFN (including NIFTP in malignant tumours) [4, 8]. In the present study, the malignancy rates for thyroid nodules diagnosed as Bethesda III and IV following resection (25 and 27.6%, respectively) are consistent with the literature. These rates may be considered to guide clinicians when deciding whether to perform a thyroidectomy, as well as to encourage pathologists to reconsider the current recommendations given by the Bethesda System for Reporting Thyroid Cytopathology. However, these results may not be generalisable to AUS/FLUS or FN/SFN cohorts, even though the rates are remarkedly similar to the rates observed in our study. In addition, other published cohorts with a smaller size have reported a malignancy risk for AUS/FLUS nodules as high as 46% [15, 17]. Differences in malignancy rates may be related to variability in randomisation, between institutions or in pathologic interpretation.

The feasible classification of thyroid nodules based on FNAC has provided an insight into the implications for histopathology, focused on the malignancy risk among thyroid lesions [18, 19]. In the present study, the rate of malignancy among patients who underwent immediate surgery was 16% for class III and 28.6% for class IV. Surprisingly, the malignancy rate following two successive FNACs increased to 45.5% for class III but did not change significantly for class IV (25%). Including all resected nodules, the rates of malignancy for all patients triaged to surgery were 25 and 27.6%, respectively. Cavalheiro et al. reported a malignancy rate of 16% among thyroid nodules classified as Bethesda category III, and 17% among those classified as Bethesda category IV [20]. In 2019, Chirayath et al. studied the malignancy rates for nodules classified as Bethesda categories III and IV in a prospective study including 176 consecutive nodules. They advised surgery for patients with a category IV diagnosis, whereas those diagnosed with category III nodules were given the option of a repeat FNA in 3 months or immediate surgery. The malignancy rates of Bethesda categories III and IV for patients triaged for immediate surgery were 54.6 and 72.4%, respectively, which are much higher than the rates reported by the ATA and by our study [21]. The main reason for this difference from our study may be the heterogeneous and subjective interpretation of Bethesda categories between pathologists/cytologists at different institutions. Also, epidemiological and geographical differences between populations should not be ignored. Our outcomes highlight an important point in clinical practice, that there may be no need to repeat the biopsy of lesions firstly diagnosed as class IV, but lesions classified as class III may need a repeated FNAC.

Similar to our findings for Bethesda categories III and IV, Cavalheiro et al. also reported that PTC cases represented a majority of the malignant thyroid neoplasms [20]. In a cohort of 4827 cytological specimens, 806 cases were classified as AUS, among whom 255 patients underwent a thyroidectomy, with a malignancy rate of 39% [22]. Mathur et al. reported that AUS subclassifications such as the “presence of focal nuclear atypia”, “focal microfollicular proliferation”, “focal Hurthle cell proliferation” and “others” were associated with malignancy rates of 54, 39, 19, and 26%, respectively. These are higher risks of malignancy than originally predicted based on The Bethesda System. Therefore, the authors recommended surgical resection for this cytological condition [22]. In another study that investigated 3080 thyroid FNACs, the malignancy rates in Bethesda categories III and IV were 17 and 25.4%, respectively [23], which are comparable to our findings. Horne et al. also subclassified 106 nodules according to microfollicular architecture (corresponding to FLUS) and nuclear atypia (corresponding to AUS), giving malignancy rates of 7 and 56%, respectively [18]. The possibility of malignant neoplasms outside the limits of the Bethesda System suggest that undetermined nodules with nuclear atypia could be at substantially higher risk for malignancy.

The mean age, gender and thyroid nodule size in the current study are comparable to other reports [8, 16, 18]. Alexander et al. studied 577 patients with undetermined nodules using a molecular classifier and reported a majority of female patients (78.2%), median age of 52.8 years and median nodule size of 2.2 cm [16]. Ho et al. studied 541 AUS thyroid nodules in patients with a median age of 54 years, 80.4% of whom were females, and the median nodule size was 1.9 cm [8]. In our study, the mean age of 155 patients classified as AUS/FLUS or FN/SFN was 52.5 years, the percentage of female patients was 85.2% and the mean size of nodules was 1.9 cm, in accordance with previous studies. Although we did not perform an analysis of the correlation of age, gender and nodule size with the malignancy rate, we believe that these results are valuable as they are consistent with the literature. Future studies should determine whether a correlation exists between the malignancy rate and demographic parameters, as the prevalence of malignancy may vary between institutions.

In Turkey, an aggressive surgical approach for nodules classified as Bethesda class III is not recommended because the primary role of resection assessment is to identify patients who do not require an operation for thyroid nodules. Thus, follow-up of suspicious nodules and repeated FNAC is usually recommended for the clinical management of thyroid nodules [24]. However, a Bethesda IV diagnosis may require a different type of management. In a study by Tepeoglu et al., the rates of malignancy for AUS/FLUS and FN/SFN were 12.7 and 35.0% for 1021 cases, respectively. Each of these diagnostic categories in Turkish patients were comparable to our findings. Thus, if a surgery is inevitable in cases diagnosed with Bethesda category IV nodules, we suggest a diagnostic lobectomy as the most aggressive approach rather than total thyroidectomy. Comparing the Bethesda System for Reporting Thyroid Cytopathology, the choice for the management of nodules may be determined by a cytopathological follow-up or molecular testing, which becomes instrumental to rule out cancer judiciously and reduce unnecessary thyroidectomies [25]. Our findings are comparable with the literature for Bethesda category III and IV nodules, the two most controversial cytological categories, giving a range of 10–30% for AUS/FLUS and 25–40% for FN/SFN based on the reviewed data [4, 8]. Use of this system is heterogeneous across institutions, and there is some degree of subjectivity when distinguishing between categories III and IV [6, 22]; therefore, it is crucial to estimate the rates of malignancy at each institution.

There are some genetic studies for presurgical differentiation of Bethesda classes III and IV to avoid the need for diagnostic surgery [26–28]. Due to the high sensitivity and accuracy, genetic analysis may be helpful in ruling out malignancy in cases of indeterminate nodules. However, there are not yet efficient and cost-effective for routine clinical use; therefore, genetic pathways for thyroid cancer are being investigated experimentally using new genetic technologies.

## Conclusion

This work provides a more precise correlation of malignancy rates with thyroid nodules classified as Bethesda categories III and IV, as our findings are comparable with the literature, giving malignancy rates ranging from 10 to 30% for category III and 25–40% for category IV. Use of the Bethesda System for Reporting Thyroid Cytopathology is heterogeneous across institutions, and there is some degree of subjectivity in the distinction between categories III and IV. Therefore, it is important to estimate the rates of malignancy at each institution.

With regard to future objectives, molecular assays are gaining importance for determining the need for surgical interventions for thyroid lesions. Gene expression assays using FNAC material may demonstrate a high predictive value in cytological undetermined thyroid nodules diagnosed as Bethesda classes III and IV.

## Data Availability

The datasets used and/or analysed during the current study available from the corresponding author on reasonable request.

## Abbreviations

BSRTC: Bethesda System for Reporting Thyroid Cytopathology
FNAC: Fine needle aspiration cytology
NIFTP: Noninvasive follicular thyroid neoplasm with papillary-like nuclear features
FLUS: Follicular lesion of undetermined significance
FN/SFN: Follicular neoplasm / suspicious for follicular neoplasm
H&E: Hematoxylin and eosin
UMP: Uncertain malignant potential
PTC: Papillary thyroid carcinoma
WDT: Well differentiated thyroid tumor
EFV: Encapsulated follicular variant
